# Supplementary material for: Multifunctional, CD44v6-Targeted ORMOSIL Nanoparticles Enhance Drugs Toxicity in Cancer Cells
Source: Nanomaterials (Basel). 2020 Feb 10;10(2):298. doi: 10.3390/nano10020298 (PMC7075197; doi:10.3390/nano10020298)
Supplement: Supplementary file 1 [file nanomaterials-10-00298-s001.pdf]

Supplementary Materials: Multifunctional, CD44v6-Targeted ORMOSIL Nanoparticles Enhance Drugs Toxicity in Cancer Cells

Lucía Morillas-Becerril [1,†], Elektra Peta [2,†], Luca Gabrielli [1], Venera Russo [2], Elisa Lubian [1], Luca Nodari [3], Maria Grazia Ferlin [4], Paolo Scrimin [1], Giorgio Palù [2], Luisa Barzon [2], Ignazio Castagliuolo [2], Fabrizio Mancin [1,*] and Marta Trevisan [2,*]

[1] Dipartimento di Scienze Chimiche, Università di Padova, via Marzolo 1, 35131 Padova, Italy; lucia.morillasbecerril@studenti.unipd.it (L.M.-B.); luca.gabrelli@unipd.it (L.G.); elisa.lubian81@gmail.com (E.L.); paolo.scrimin@unipd.it (P.S.)
[2] Department of Molecular Medicine, University of Padova, via Gabelli 63, 35121 Padova, Italy; elektra.peta@unipd.it (E.P.); verarusso87@gmail.com (V.R.); giorgio.palu@unipd.it (G.P.); luisa.barzon@unipd.it (L.B.); ignazio.castagliuolo@unipd.it (I.C.)
[3] ICMATE-CNR, Area della Ricerca di Padova, C.so Stati Uniti 4, 35127 Padova, Italy; luca.nodari@cnr.it
[4] Dipartimento di Scienze Farmaceutiche, Università di Padova, Via Marzolo 5, 35131 Padova, Italy; mariagrazia.ferlin@unipd.it
* Correspondence: fabrizio.mancin@unipd.it (F.M.); marta.trevisan@unipd.it (M.T.)
† These authors contributed equally to this work.

Characterization of the nanoparticles

Fluorescamine test

A fluorescamine reagent was used to quantify the number of free amino groups, through extrapolation from a calibration curve, present in the samples after conjugation with antibody and hyaluronic acid.

Equation (1). Calibration curve with fluorescamine.

$$y = 87.236 + 15.058x$$

Table S1. Fluorescence emission of Ab-CD44v6-NPs and HA-NPs.

	**Fluorescence**	**[NH_2] (mM)**
NH_2-NPs	1397.2825	4.35
Ab-CD44v6-NPs	247.6037	0.53
HA-NPs	440.0449	1.17

A total of 87% of free amino groups were obtained before conjugation for NH2-NPs, while 5% and 11% of free amino groups were obtained after conjugation with antibody Ab-CD44v6 and after conjugation with HA, respectively.

Ellmann test

Ellman's reagent (5,5'-dithiobis-(2-nitrobenzoic acid) was used to quantify the number of thiol groups per protein in the derivatized antibodies using Equations (2)–(4) [65,66].

Equation (2). Calculation of molar concentration of protein

$$[Protein]_{280} = \frac{Absorbance_{280}}{\varepsilon_{protein}};\ being\ \varepsilon_{HSA} = 35700\ M^{-1}cm^{-1}\ and\ being\ \varepsilon_{CD44} = 210000\ M^{-1}cm^{-1}$$

Equation (3). Calculation of molar concentration of thiol groups.

$$[SH] = \frac{Absorbance\ protein_{412} - Absorbance\ blank_{412}}{\varepsilon_{DTNB}}; being\ \varepsilon_{DTNB} = 136000\ M^{-1}cm^{-1}$$

Equation (4). Calculation of derivatization.

$$SH = \frac{SH\ molar\ concentration}{Protein\ molar\ concentration}$$

Table S2. Absorbance of HAS-NPs and Ab-CD44v6-NPs.

	$\boldsymbol{Absorbance\ blank_{412}}$	$\boldsymbol{Absorbance_{280}}$	$\boldsymbol{Absorbance_{412}}$
HAS-NPs	0.1076	1.0426	0.5774
Ab-CD44v6-NPs	0.1076	1.0445	0.4273

A total of 1.2 thiol groups per albumin were obtained for the control sample, and 4.7 thiol groups per Ab-CD44v6 antibody were obtained.

1. Rhod-NPs

Table S3. Summary table of Rhod-NPs.

	**Hydrodynamic diameter (nm)**	**PDI**	**[Dye]*, (µM)**	**[NPs]**, (mg/mL)**
NPs	97	0.019	10.11	2.46

* Absorption spectroscopy was used to determine the concentration of dye loaded; therefore, a calibration curve was performed in a solvent mixture which could mimic the internal environment of the nanoparticles.
** [NPs] calculated performing a TGA analysis on 100 µL of NP solution.

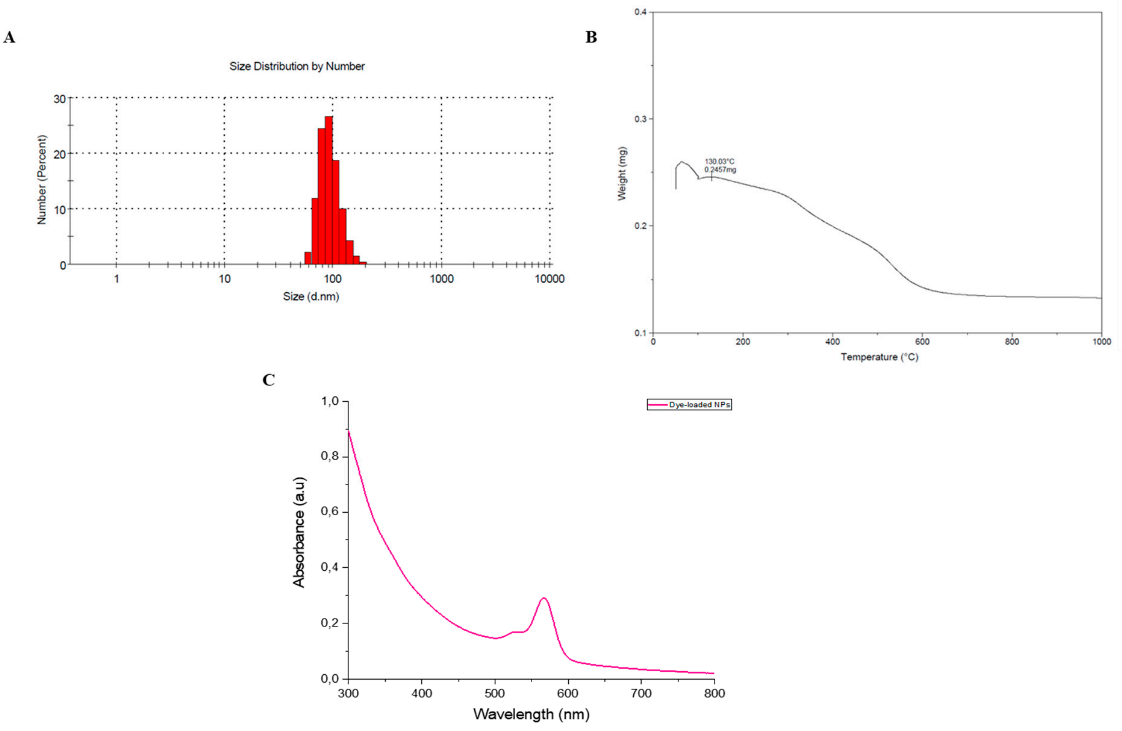

Figure S1. DLS distribution (**A**), TGA analysis (**B**) and UV-Vis spectrum (**C**) of Rhod-NPs.

2. MG2477-NPs

Table S4. Summary table of MG2477-NPs.

	Hydrodynamic diameter (nm)	PDI	[Drug]*, (μM)	[NPs]**, (mg/mL)
NPs	140	0.095	46.5	4.30

* Fluorescence emission spectroscopy was used to determine the concentration of drug loaded; therefore, a calibration curve was performed in a solvent mixture which could mimic the internal environment of the nanoparticles.
** [NPs] calculated performing a TGA analysis on 100 μL of NP solution.

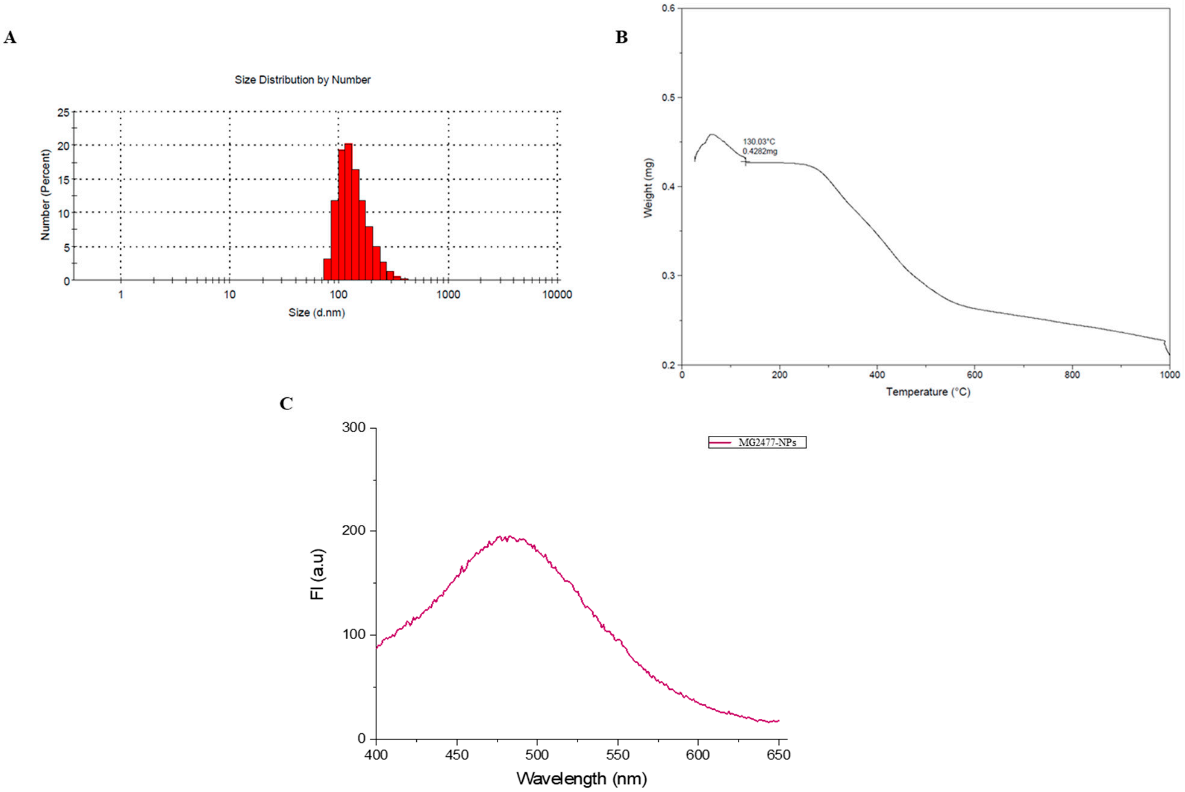

Figure S2. DLS distribution (**A**), TGA analysis (**B**) and fluorescence spectrum (**C**) of MG2477-NPs.

3. Ab-CD44-MG2477-NPs

Table S5. Summary table of Ab-CD44v6-MG2477-NPs.

	Hydrodynamic diameter (nm)	PDI	Corediameter, TEM (nm) (mean ± SD)	[Drug]*, (μM)	[NPs]**, (mg/mL)
Ab-CD44v6^{1x}-MG2477-NPs	104	0.195	110 ± 60	1.20	0.81
Ab-CD44v6^{10x}-MG2477-NPs	98	0.207	90 ± 30	1.30	0.79

* Fluorescence emission spectroscopy was used to determine the concentration of drug loaded; therefore, a calibration curve was performed in a solvent mixture which could mimic the internal environment of the nanoparticles.
** [NPs] calculated performing a TGA analysis on 100 μL of NP solution. Conjugation of nanoparticles was performed in PBS:ETDA, so nanoparticle concentration was obtained through subtraction with the value obtained for the solvent mixture.

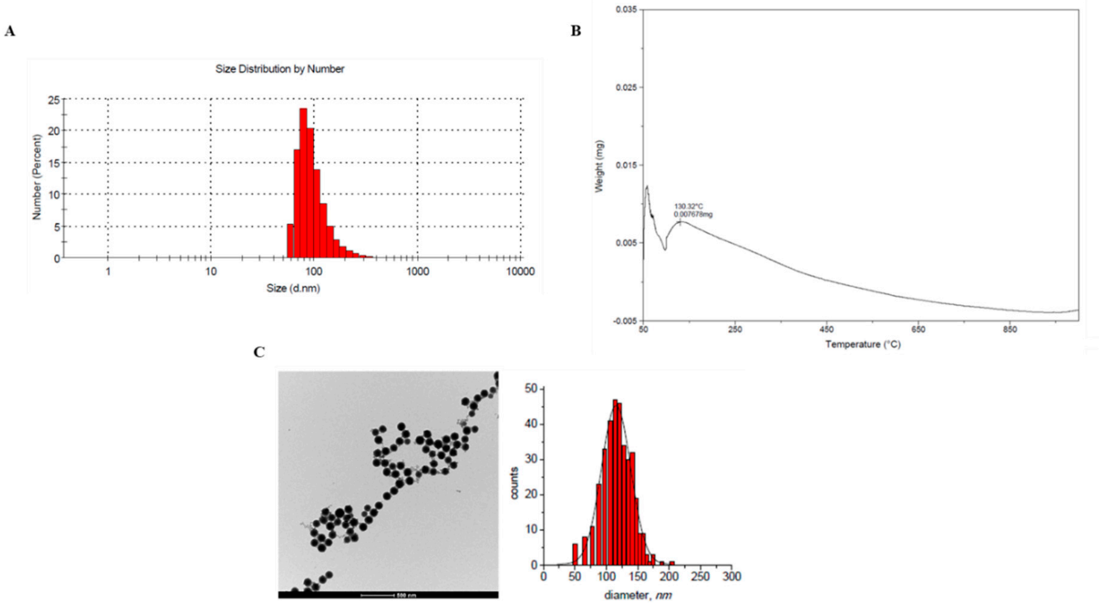

Figure S3. DLS distribution (**A**), TGA analysis (**B**), TEM image size distribution and fitting curve parameters (**C**, average diameter = 110 nm, σ = 60 nm) of Ab-CD44v6[1x]-MG2477-NPs.

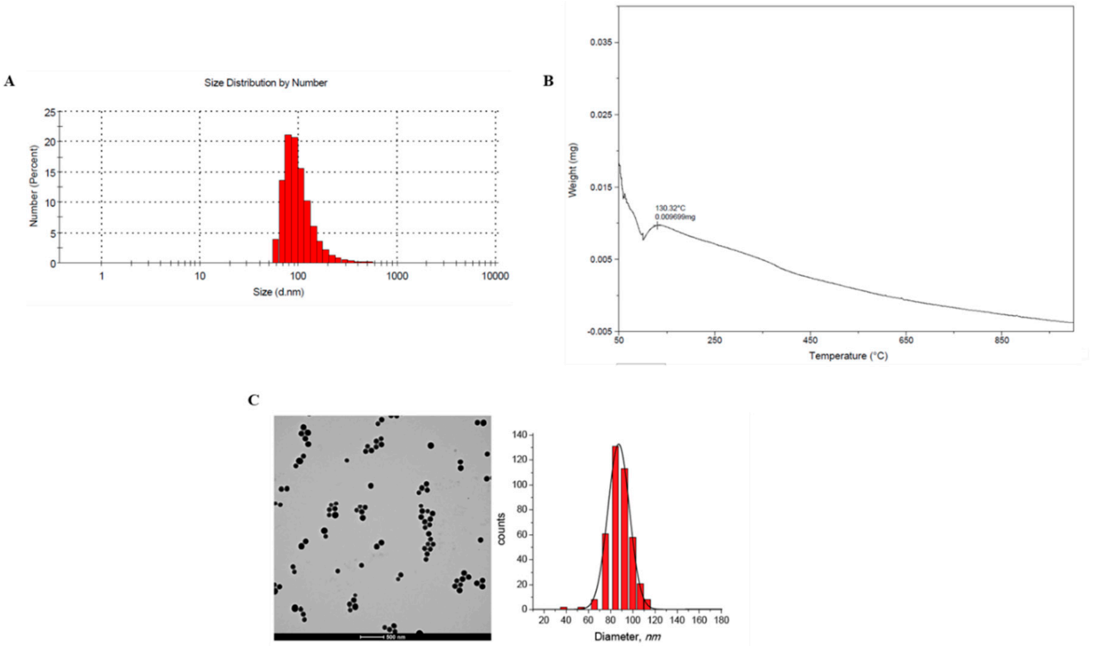

Figure S4. DLS distribution (**A**), TGA analysis (**B**) and TEM image size distribution and fitting curve parameters (**C**, average diameter = 90 nm, σ = 30 nm) of Ab-CD44v6[10x]-MG2477-NPs.

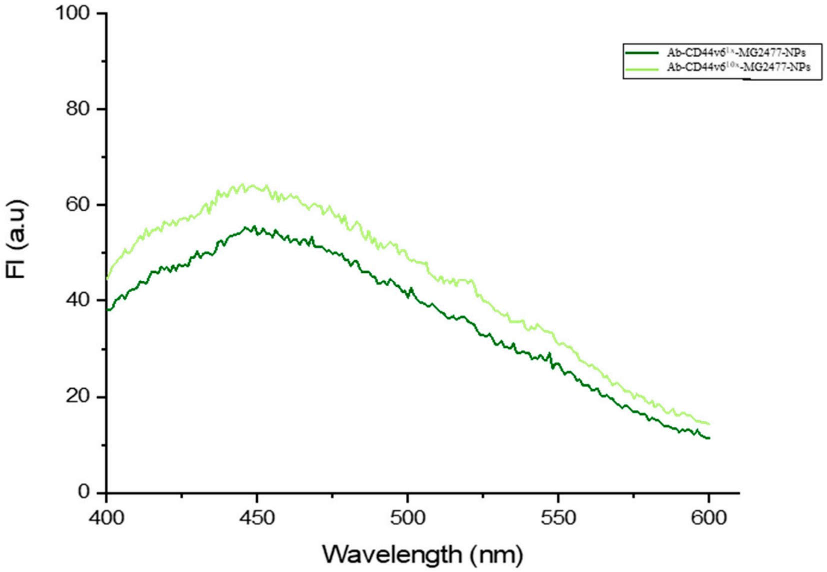

Figure S5. Fluorescence spectrum of Ab-CD44v6-MG2477-NPs ($\lambda_{exc} = 350\ nm, slit_{exc} = 5\ ,\ slit_{em} = 10$).

4. HA-MG2477-NPs

Summary table:

Table S6. Summary table of HA-MG2477-NPs.

	Hydrodynamic diameter (nm)	PDI	Corediameter, TEM (nm) (mean ± SD)	[Drug]*, (µM)	[NPs]**, (mg/mL)
11.5 kDa HA^{1x}-MG2477-NPs	147	0.019	153 ± 38	5.73	2.98
11.5 kDa HA^{10x}-MG2477-NPs	135	0.062	136 ± 23	9.57	4.22
22.5 kDa HA^{1x}-MG2477-NPs	134	0.074	130 ± 30	4.65	2.80
22.5 kDa HA^{10x}-MG2477-NPs	125	0.108	127 ± 22	7.79	3.99

* Fluorescence emission spectroscopy was used to determine the concentration of drug loaded; therefore, a calibration curve was performed in a solvent mixture which could mimic the internal environment of the nanoparticles.

** [NPs] calculated performing a TGA analysis on 100 µL of respective NP solution. Conjugation of nanoparticles was performed in PBS, so nanoparticle concentration was obtained through subtraction with the value obtained for the solvent.

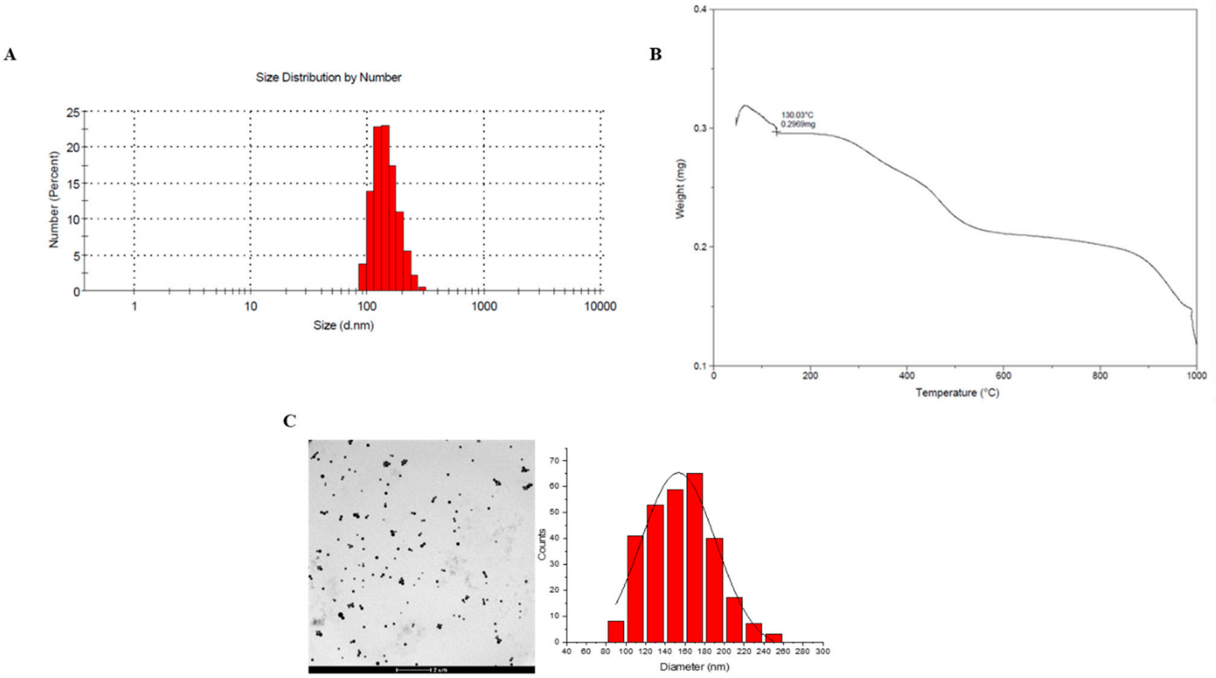

Figure S6. DLS distribution (**A**), TGA analysis (**B**), TEM image size distribution and fitting curve parameters (**C**, average diameter = 153 nm, σ = 38 nm) of 11.5 kDa HA^{1x}-MG2477-NPs.

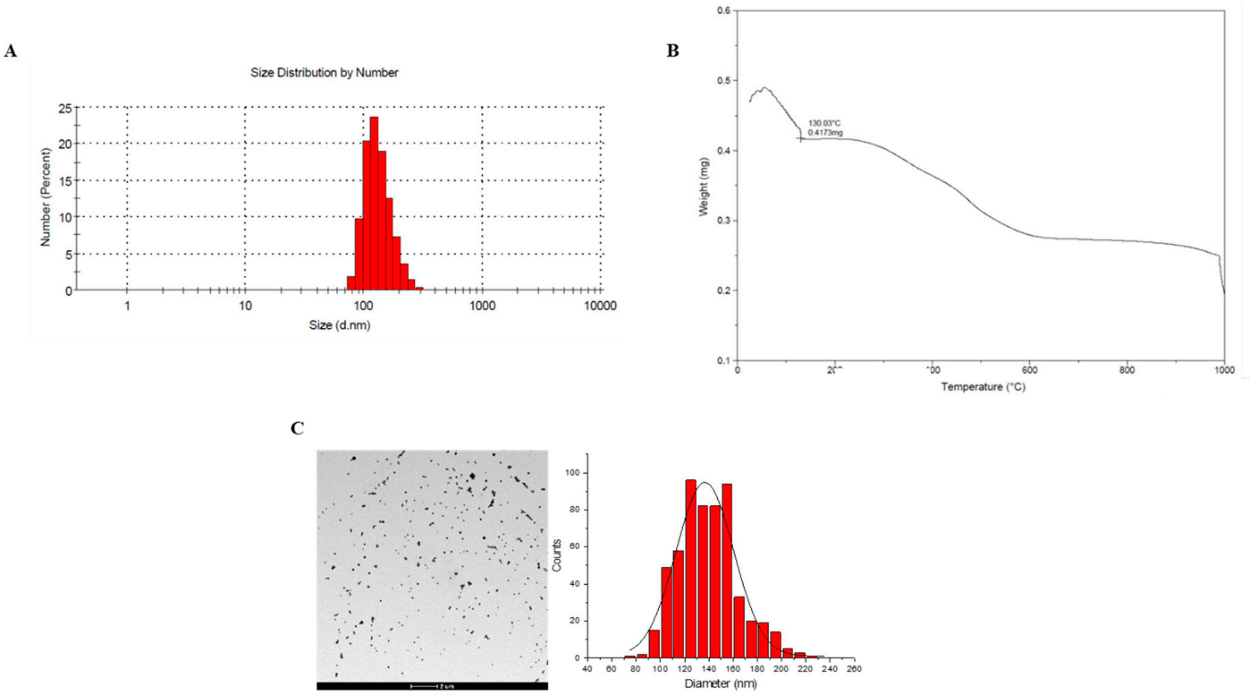

Figure S7. DLS distribution (**A**), TGA analysis (**B**), TEM image size distribution and fitting curve parameters (**C**, average diameter = 136 nm, σ = 23 nm) of 11.5 kDa HA^{10x}-MG2477-NPs.

A

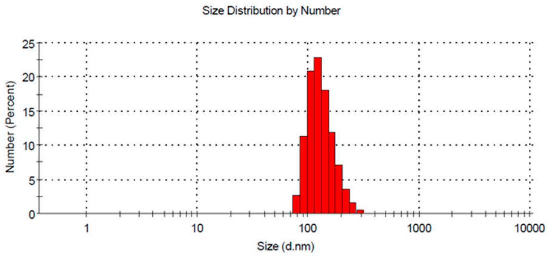

B

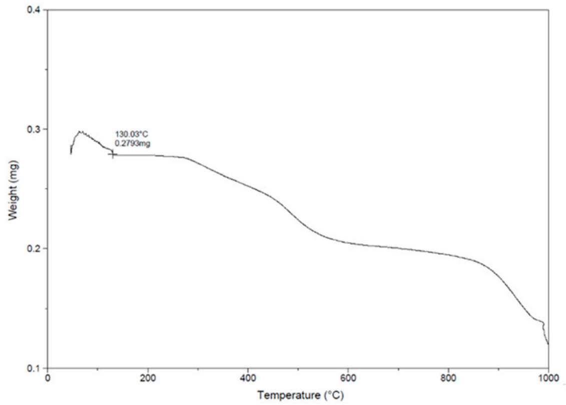

C

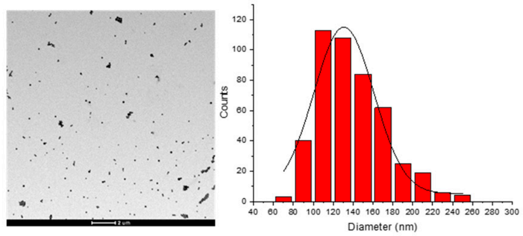

Figure S8. DLS distribution (**A**), TGA analysis (**B**), TEM image size distribution and fitting curve parameters (**C**, average diameter = 130 nm, σ = 30 nm) of 22.5 kDa HA^{1x}-MG2477-NPs.

A

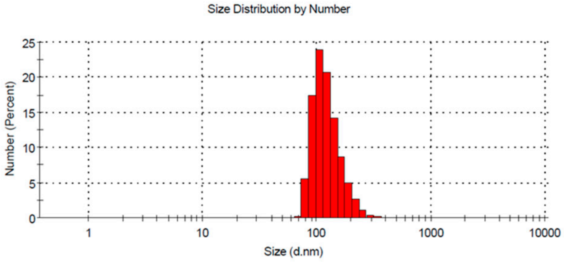

B

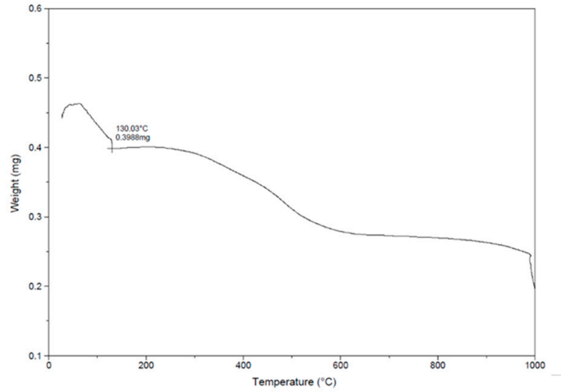

C

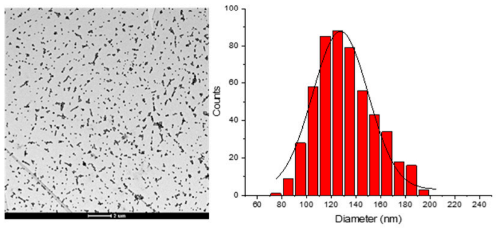

Figure S9. DLS distribution (**A**), TGA analysis (**B**), TEM image size distribution and fitting curve parameters (**C**, average diameter = 127 nm, σ = 22 nm) of 22.5 kDa HA^{10x}-MG2477-NPs.

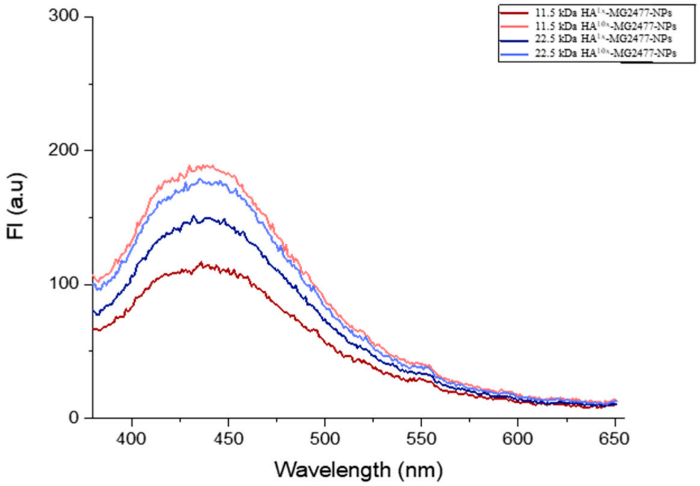

Figure S10. Fluorescence spectrum of HA-MG2477-NPs ($\lambda_{exc} = 350\ nm, slit_{exc=}slit_{em} = 5$).

5. Infrared spectroscopy of HA-MG2477-NPs

Four spectra were collected on different aggregates deposited on the glass-slide. All the spectra show a strong absorption band in the 1200–900 cm⁻¹ range and by a broad absorption in the ν(OH) region.

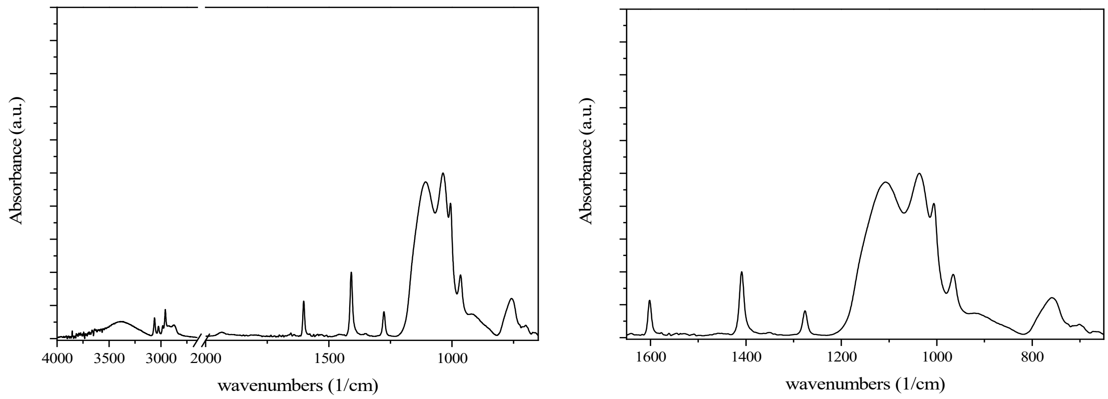

Figure S11. FTIR spectrum of nanoparticles before conjugation with hyaluronic acid.

After conjugation with hyaluronic acid, the signal of AI and AII are clearly detectable at 1652 and 1543 cm^{-1}, respectively. The signal at 1743 cm^{-1} can be associated to the ν(C=O) stretching in a saturated aliphatic acid.

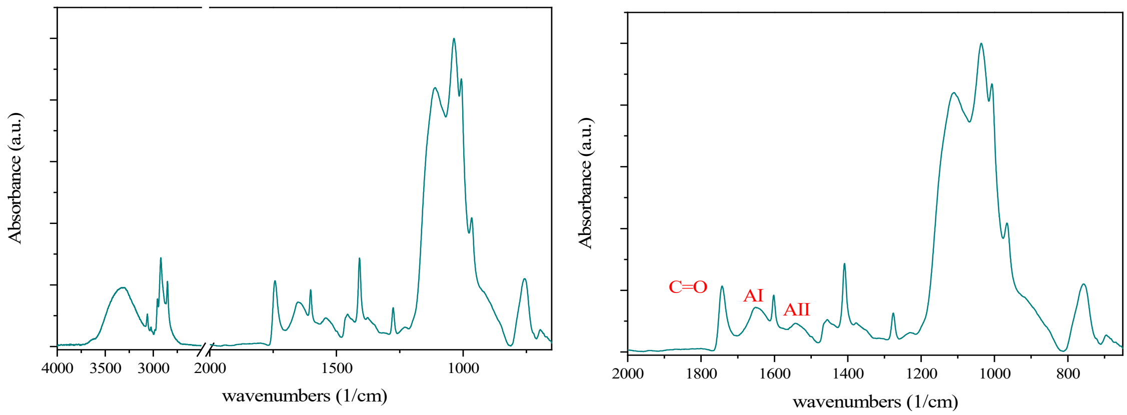

Figure S12. FTIR spectrum of nanoparticles after conjugation with hyaluronic acid.

Titration of antibody (Ab-CD44v6) conjugated to NPs was performed by SDS-PAGE

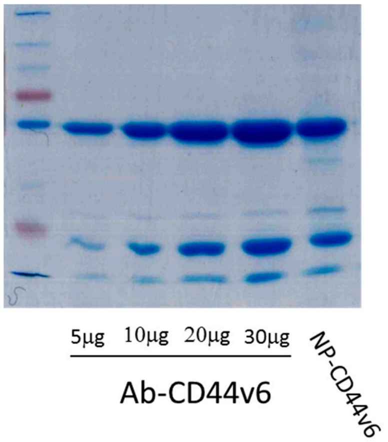

Figure S13. Representative titration of antibody (Ab-CD44v6) conjugated to NPs was performed by SDS-PAGE, by loading into the gel a fixed quantity of Ab-CD44v6-Rho-NPs together with different known concentrations of IgG Ab-CD44v6 and by extrapolating the concentration by comparison with the calibration curve after plotting band density.

Analysis of the expression of CD44v6 in HEK-293A-CD44v6 and HeLa-CD44v6:

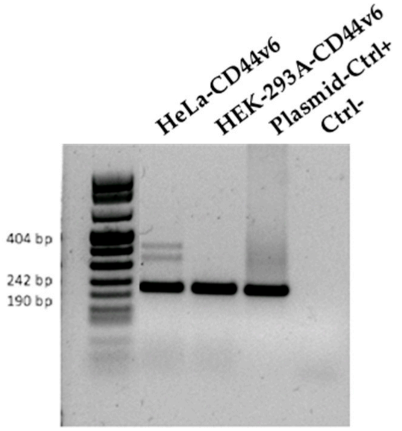

Figure S14. RNAs were isolated from transfected HEK-293A-CD44v6 and HeLa-CD44v6 cells, reverse transcribed and CD44v6 was amplified by RT-PCR as previously described. Amplicons of CD44v6 (202bp) were loaded on a 1.5% agarose gel.

Ab-conjugated-NPs binding analysis by fluorescence microscopy

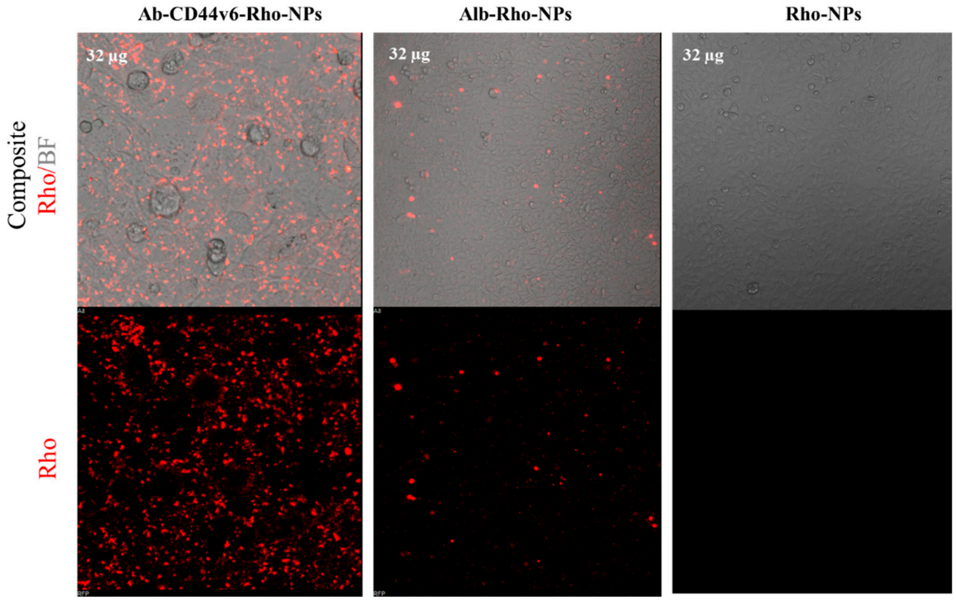

Figure S15. Ab-CD44v6-NPs binding analysis by fluorescence microscopy. HEK-293A-CD44v6 were incubated with 32 µg of unconjugated Rho-NPs, albumin conjugated NPs (Alb-Rho-NPs) and Ab-CD44v6-conjugated NPs (Ab-CD44v6-Rho-NPs), for 1 h at RT. Cells were then washed multiple times to eliminate unbounded NPs and observed at a fluorescence microscope; Rho signal (in red), BF (bright field) signal in grey; Magnification 10×.

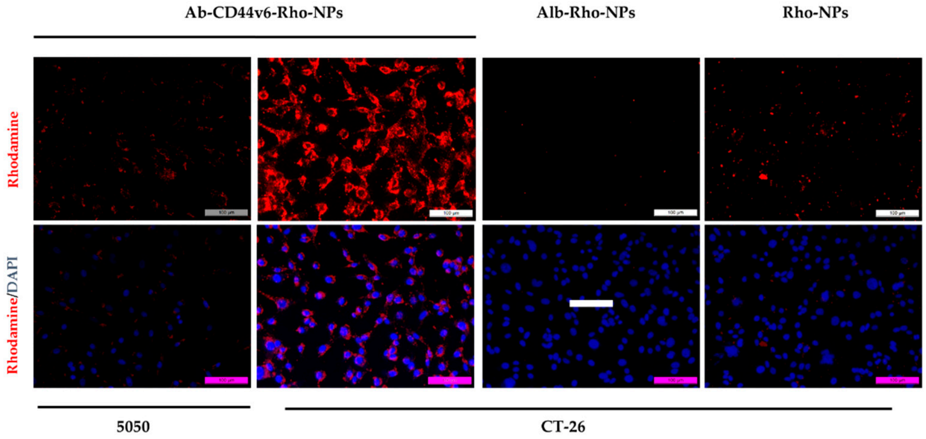

Figure S16. Internalization of Ab-CD44v6-Rho-NPs in CT-26 cells and bovine 5050 cells. CT-26 and 5050 cells were incubated with Ab-CD44v6-Rho-NPs 0.1 mg/ml for 4 h at 37 °C and then washed several times to eliminate unbound NPs. Cells were then observed at a fluorescent microscope to analyze internalized NPs. Scale bars: 100 µm.

Internalization of Ab-CD44v6-Rho-NPs in HEK-293A-CD44v6 cells:

Non-specific competition assay: To verify if cellular uptake of conjugated NPs into cells is due to antibody–antigen interaction rather than a non-specific binding, a competition assay was performed using an excess of free γ-globulins or of anti-HDAC11 Ab (for 1 h at 37 °C), before internalization of NPs. As shown in Figure S17, a treatment with non-specific Abs did not cause any reduction of fluorescence intensity, demonstrating that endocytosis of conjugated NPs cannot be blocked by non-specific interactions.

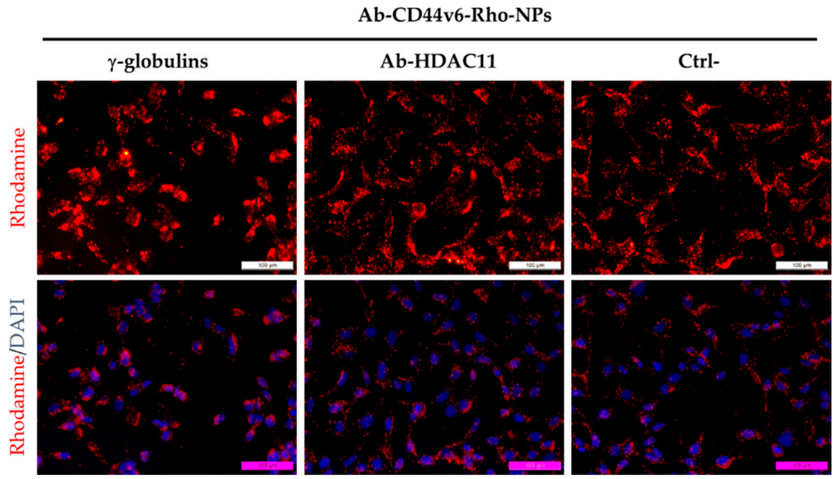

Figure S17. Non-specific competition assay: internalization of Ab-CD44v6-Rho-NPs in HEK-293A-CD44v6 upon incubation with specific γ-globulins and anti-HDAC11. HEK-293A-CD44v6 were treated with an excess of γ-globulins or anti-HDAC11 antibody for 1h at 37 °C. Cells were then treated with Ab-CD44v6-Rho-NPs for 4 h at 37 °C and washed to eliminate unbound NPs. A fluorescent microscope was used to analyze internalized NPs (Rhodamine B signal). DAPI: 4′,6-diamidino-2-phenylindole; Scale bars: 100 µm.

Internalization of HA-Rho-NPs in HEK-293A-CD44v6 cells:

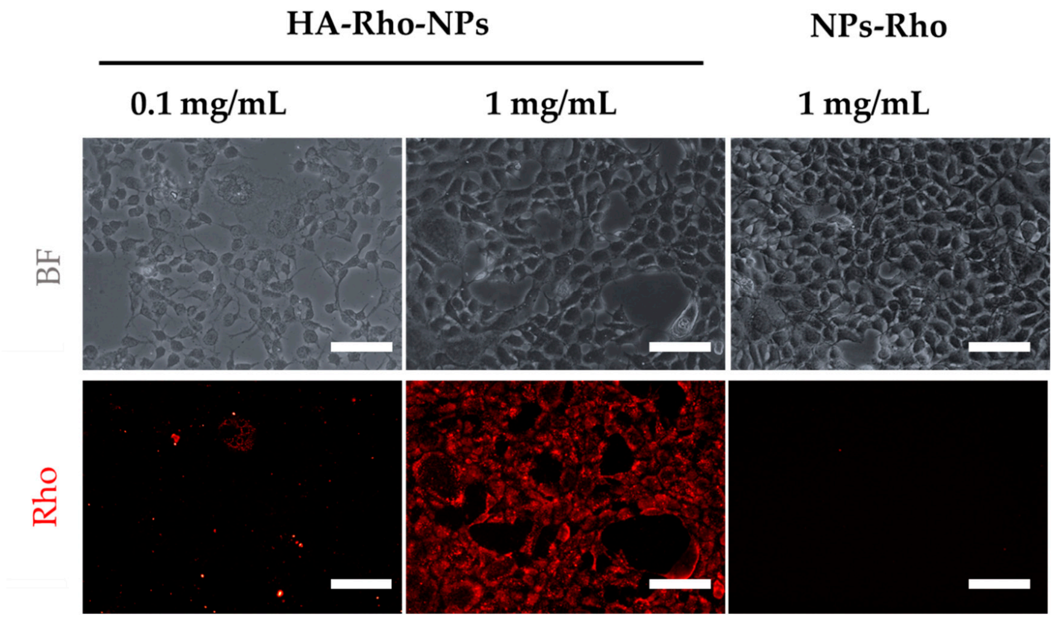

Figure S18. Internalization of HA-Rho-NPs in HEK-293A-CD44v6 cells. Cells were incubated with different concentrations of HA-Rho-NPs and with Rho-NPs (1 mg/mL) for 4 h at 37 °C and then washed several times to eliminate unbound NPs. Cells were then observed at a fluorescent microscope to analyze internalized NPs. BF: Bright Field; Rho: Rhodamine signal. Scale bars: 100 µm.

Cytotoxicity test of Ab-CD44v6-MG2477-NPs in HEK-293A-CD44v6

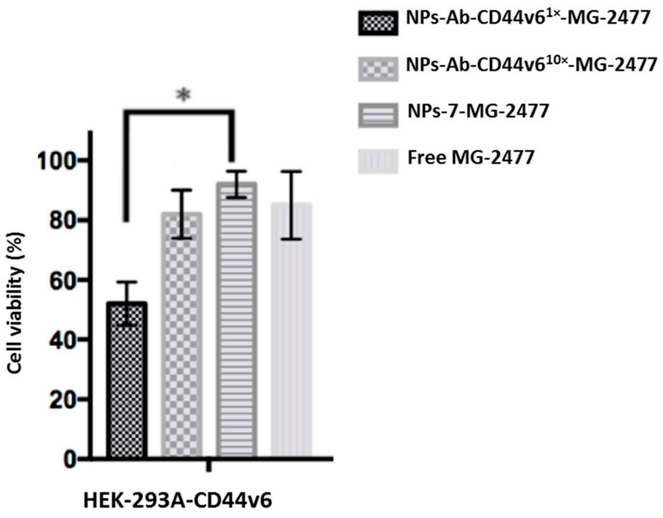

Figure S19. Analysis of the cytotoxicity of Ab-CD44v6-MG2477-NPs loaded with MG2477 (0.01 μM) in HEK239A-CD44v6, 72 h post treatment. Ab-CD44v6^{10x}-MG2477-NPS and Ab-CD44v6^{1x}-MG2477-NPs: NPs conjugated with a greater or a lower amount of antibody (ratio of NPs/Ab 1:10 and 1:1 respectively). *$p \leq 0.05$.

References

[64] Ellman, G.L. Tissue Sulphidryl Groups. *Arch. Biochem. Biophys*. **1959**, *82*, 70–77.

[65] Bulaj, G.; Kortemme, T.; Goldenberg, D.P. Ionization−Reactivity Relationships for Cysteine Thiols in Polypeptides. *Biochemistry*, **1998**, *37*, 8965–8972.
